# Phenotypic, immunologic, and clinical characteristics of patients with nontuberculous mycobacterial lung disease in Korea

**DOI:** 10.1186/1471-2334-13-558

**Published:** 2013-11-25

**Authors:** Ae-Ra Lee, Jinwoo Lee, Sun-Mi Choi, Moon-Woo Seong, Sung A Kim, Myoungsil Kim, Kyung Ok Chae, Ji Sun Lee, Jae-Joon Yim

**Affiliations:** 1 Division of Pulmonary and Critical Care Medicine, Department of Internal Medicine, Seoul National University College of Medicine, 101 Daehak-ro, Jongno-gu, Seoul 110-744, Republic of Korea; 2 Department of Laboratory Medicine, Seoul National University College of Medicine, 101 Daehak-ro, Jongno-gu, Seoul 110-744, Republic of Korea

**Keywords:** Bronchiectasis, Clinical characteristics, Immunopathogenesis, Nontuberculous mycobacterial lung disease, Phenotype

## Abstract

**Background:**

This study aimed to elucidate the phenotypic, immunologic, and clinical characteristics of Korean patients with nontuberculous mycobacterial (NTM) lung disease and compare them with non-NTM bronchiectasis (BE) patients.

**Methods:**

We prospectively recruited patients between 20 and 80 years of age who had nodular BE type NTM lung disease. Phenotypic, immunologic, and clinical characteristics were evaluated through physical examination, laboratory tests, pulmonary function tests, and radiographic examinations. Questionnaires were also answered. The results of the evaluations were compared with the results of non-NTM BE patients.

**Results:**

A total of 84 patients with NTM lung disease and 47 non-NTM BE patients participated in the study. *Mycobacterium avium* complex lung disease and *M. abscessus* lung disease were most common. Patients with NTM lung disease had lower body mass index than non-NTM BE patients. Scoliosis was observed more frequently in patients with NTM lung disease than in non-NTM BE patients.

**Conclusions:**

Significant similarities were seen between Korean patients with NTM lung disease and patients from other countries. Differences in phenotypic and clinical characteristics between NTM lung disease and non-NTM BE patients suggest differences in the immunopathogenesis of NTM lung disease and non-NTM BE.

**Trial registration information:**

ClinicalTrials.gov Registration number; NCT01616745

## Background

Nontuberculous mycobacteria (NTM) are environmentally ubiquitous organisms, and rarely cause disease in healthy individuals. Rates of NTM isolation, as well as the number of patients with NTM lung disease, have been increasing worldwide [1]; however, the fact that only a small number of people contract NTM despite ubiquitous exposure suggests the presence of identifiable risk factors associated with NTM infection.

While the immunopathogenesis of NTM lung disease remains largely unknown, certain phenotypic and immunologic characteristics of patients with NTM lung disease have been observed. A single nucleotide polymorphism in TLR2 has been linked to NTM lung diseases [2], and IFN-γ and IL-10 secretions have been reported to be suppressed among patients with NTM lung disease [3]. Furthermore, patients with NTM lung disease tend to be taller and leaner on average, with relatively high frequencies of scoliosis, pectus excavatum, and mitral valve prolapse [4].

While the incidence of tuberculosis has been in steady decline across South Korea, the rate of NTM isolation has increased rapidly [5]. The aim of this study was to elucidate the phenotypic, immunologic, and clinical characteristics of Korean patients with NTM lung diseases through comparisons with non-NTM bronchiectasis (BE) patients.

## Methods

### Study cohort

Beginning July 1, 2011 we prospectively recruited patients between 20 and 80 years of age at Seoul National University Hospital, Seoul, South Korea, who met the diagnostic criteria for NTM lung disease set forth by the American Thoracic Society [6]. Patients previously treated for NTM lung disease were excluded from this study. NTM patients with nodular BE types were included but those with upper lobe cavitary types were excluded. All patients provided written informed consent before enrollment. The study protocol was approved by the Institutional Review Board of Seoul National University Hospital. The clinical trial registration number is NCT 01616745 (www.ClinicalTrials.gov).

### Control group

Beginning January 1, 2012 we began recruiting patients ≥ 20 years old diagnosed with BE in the absence of NTM infection (non-NTM) to serve as a control group. BE was diagnosed based on low dose computed tomography (CT) findings that included dilatation of an airway lumen, rendering it more than 1.5 times the width of a nearby vessel, lack of tapering of an airway toward the periphery, varicose constrictions along airways, and ballooned cysts at the end of a bronchus [7]. Two separate sputum mycobacterial cultures were performed to exclude patients with active NTM infections. The median interval between the two cultures was 24 months (interquartile range: 9–55 months).

### Physical examinations

Physical examinations were performed by board-certified physicians. Height and weight were measured by a team of two nurses.

### Microbiological tests

Sputum was collected for bacterial and mycobacterial cultures. Samples of sputum were homogenized by incubation at 37°C for 15 min with an equal volume of 0.1% dithiothreitol (Sputolysin; Calbiochem Corp., San Diego, CA, USA). Homogenized sputum was sequentially diluted and placed in phosphate-buffered saline and plated on blood, chocolate, and MacConkey agar plates. Sputum isolates were classified as potential pathogens or as normal flora. Potential pathogens were *Haemophilus influenzae*, *Moraxella catarrhalis*, *Streptococcus pneumoniae*, *Pseudomonas aeruginosa*, *Staphylococcus aureus*, and other gram-negative rods; other bacterial species were classified as normal flora [8].

Sputum and bronchial washing fluid were decontaminated with 4% sodium hydroxide, homogenized, and concentrated by centrifugation at 3000 × *g* for 20 min. The specimens were stained using the Ziehl–Nielsen method [6]. Concentrated specimens were cultured in 3% Ogawa medium and observed weekly for 9 weeks after inoculation. Following isolation of a suspected mycobacterial species, confirmation of NTM was performed by analyzing the sequences of three genes; *16S rRNA*, *rpoB* and *tuf*. Polymerase chain reaction and subsequent sequencing were performed, and the resulting sequences were compared with the reference database using basic local alignment search tools. Mycobacterial species were identified using *16S rRNA* sequences, using the algorithm described in Clinical and Laboratory Standards Institute guideline MM18-A [9].

### Laboratory tests

Laboratory tests consisted of the following: leukocyte count including differential counts, hematocrit, hemoglobin, platelet count, total cholesterol, total protein, albumin, total and direct bilirubin, alkaline phosphatase, aspartate aminotransferase, alanine aminotransferase, blood urea nitrogen, creatinine, electrolytes, erythrocyte sedimentation rate, C-reactive protein, fluorescent antinuclear antibody test (FANA), rheumatoid factor, serum immunoglobulins (IgG, IgA, IgM), and IFN-γ release assay (IGRA).

### Pulmonary function tests and radiographic examination

Pulmonary function tests, including forced expiratory volume at 1 second (FEV1), forced vital capacity (FVC), FEV1/FVC ratio, and diffusing capacity (DLCO) were performed. Simple posterior–anterior chest radiography, paranasal sinus radiography, and CT of the chest were carried out. Radiographic findings on CT scans were evaluated with regard to the presence of cavitations, nodule, and bronchiectasis. The anatomical distributions were also analyzed. Lesions were classified as showing either upper lobe cavitary disease or nodular bronchiectatic disease by radiographic type. When the disease did not belong to either the upper lobe cavitary form or the nodular bronchiectatic form, it was categorized as unclassifiable. The extent of bronchiectasis was scored in each of the six lobes (right upper lobe, right middle lobe, right lower lobe, upper division of the left upper lobe, lingular division of the left upper lobe, left lower lobe) according to the proportion of lung involvement. Extent scores ranged from 0 to 18; 0 if < 25%, 1 if 25–49%, 2 if 50–74%, 3 if ≥ 75% [10]. Scoliosis was determined from the posterior–anterior chest radiograph. Pectus excavatum was determined from CT scans of the chest using the Haller index and defined as a Haller index greater than 3.5 [11].

### Questionnaires

All participants were asked to complete the St. George’s respiratory questionnaire and Hospital Anxiety and Depression Scale (HADS) questionnaire. The HADS is a 14 item questionnaire measuring levels of anxiety (HADS-A, seven items) and depression (HADS-D, seven items). Each item is scored from 0–3; a cut-off point of 8 out of 21 is suggested for both the anxiety and depression sections [12].

### Analysis

Baseline characteristics were summarized using descriptive statistics such as proportion, median, and interquartile range. Student’s *t*-tests and Mann–Whitney *U*-tests were used for comparison of continuous variables. Categorical variables were compared using chi-square or Fisher’s exact tests, as appropriate. A *P*-value of ≤ 0.05 was considered to indicate statistical significance. All statistical analyses were performed using SPSS 17.0 (SPSS Inc., Chicago, IL, USA).

## Results

### Patient characteristics

A total of 127 patients with NTM lung disease were enrolled in the study, with eight patients withdrawing consent prior to study completion. Among them, 84 patients with nodular BE type NTM lung disease were included in the study. In addition, 50 patients with non-NTM BE were initially enrolled in the study; three patients withdrew consent. The median ages were 67 and 64 years for NTM lung disease and non-NTM BE groups, respectively (*P* = 0.16). Of the patients with NTM lung disease, 54 (64.3%) were female, compared with 29 (61.7%) in the non-NTM BE group. Among comorbidities, cancer was more common among the NTM lung disease group (21.4% *vs*. 6.4%, *P* = 0.03) (Table 1). Weight loss was more common among the NTM lung disease group although it failed to reach the conventional *P*-value of 0.05. (15.5% *vs*. 4.3%, *P* = 0.08) (Table 1).

**Table 1:** **Baseline characteristics of 84 patients with NTM lung disease and 47 non**-**NTM BE patients**

	**NTM lung disease patients**	**Non-NTM BE patients**	***P *** **value**
	**(N = 84)**	**(N = 47)**	
Age (year), median (IQR)	67 (57,75)	64 (58,70)	0.16
Female	54 (64.3%)	29 (61.7%)	0.85
Never smoker	57 (67.9%)	35 (74.5%)	0.55
BCG scar	170 (83.3%)	40 (85.1%)	1.00
Past medical history			
TB	23 (27.4%)	20 (42.6%)	0.08
Measles	15 (17.9%)	15 (31.9%)	0.08
Pertussis	4 (4.8%)	4 (8.5%)	0.46
Comorbidities			
^a^Sinusitis	26 (31.0%)	15 (31.9%)	1.00
COPD	17 (20.2%)	17 (37.0%)	0.06
Asthma	3 (3.6%)	1 (2.2%)	1.00
Gastroesophageal reflux disease	15 (17.9%)	11 (23.4%)	0.50
Diabetes mellitus	8 (9.5%)	1 (2.1%)	0.16
Rheumatoid arthritis	9 (10.7%)	2 (4.3%)	0.33
Cancer	18 (21.4%)	3 (6.4%)	0.03
Symptoms			
Cough	25 (29.8%)	16 (34.0%)	0.70
^b^Dyspnea	6 (7.1%)	6 (12.8%)	0.40
Sputum	39 (46.4%)	29 (61.7%)	0.10
Fever	16 (19.0%)	12 (25.5%)	0.38
Hemoptysis	19 (22.9%)	17 (36.2%)	0.15
Post nasal drip	25 (29.8%)	20 (42.6%)	0.18
Weight loss	13 (15.5%)	2 (4.3%)	0.08

*Abbreviations*: *NTM* nontuberculous mycobacteria, *BE* bronchiectasis, *TB* tuberculosis, *IQR* interquartile range, *COPD* chronic obstructive pulmonary disease.

^a^Diagnosis of sinusitis was based on self-reporting.

^b^Dyspnea was defined as modified Medical Research Council score ≥ 2.

### Microbiology analysis of patient sputum

Of the 84 patients with NTM lung disease, 47 (60.0%) were infected with species belonging to the *Mycobacterium avium* complex; 11 (13.1%) were infected with *M. abscessus* complex species. Multiple NTM species were isolated in 20 (23.8%) patients (Table 2). Bacterial colonization by non-mycobacterial species was observed in 17 (20.2%) and 12 (25.5%) of NTM lung disease and non-NTM BE patients, respectively (*P* = 1.00). The trend that *P. aeruginosa* was more commonly isolated from non-NTM BE patients (5 patients, 10.6%) than NTM patients (2 patients, 2.4%) was observed (*P* = 0.10) (Table 3).

**Table 2:** Mycobacterial species isolated from 84 patients with NTM lung disease

**The number of patients with isolation of single species**	**64/84**	**(76.2%)**	
*Mycobacterium avium* complex	47	(60.0%)	
*M. avium*		27	(32.1%)
*M. intracellulare*		18	(21.4%)
Mixed		2	( 2.4%)
*M. abscessus* complex	11	(13.1%)	
*M. abscessus*		7	( 8.3%)
*M. massiliense*		2	( 2.4%)
Mixed		2	( 2.4%)
*M. chimaera*	1	(1.2%)	
*M. kansasii*	1	(1.2%)	
*M. fortuitum* complex	1	(1.2%)	
*M. lentiflavum*	1	(1.2%)	
*M. senegalense* or *conceptionense*	1	(1.2%)	
Unidentified	1	(1.2%)	
**The number of patients with isolation of multiple species**	**20/84**	**(23.8%)**	
*M. avium* complex and others	6	(7.1%)	
* M. avium* and *M. chelonae*		1	(1.2%)
* M. avium* and *M. chimaera*		1	(1.2%)
* M. avium* and *M. fortiutum*		1	(1.2%)
* M. avium* and *M. fortiutum* and *M. kansasii*		1	(1.2%)
* M. intracellulare* and *M. chitae*		1	(1.2%)
* M. intracellulare* and *M. fortuitum*		1	(1.2%)
*M. abscessus* complex and others	5	(6.0%)	
* M. abscessus* and *M. chelonae*		1	(1.2%)
* M. abscessus* and *M. conceptionense*		1	(1.2%)
* M. abscessus* and *M. bolletii*		2	(2.4%)
* M. bolletii* and *M. lentiflavum*		1	(1.2%)
*M. avium* complex and *M. abscessus* complex	7	(8.3%)	
* M. avium* and *M. absecessus*		4	(4.8%)
* M. avium* and *M. massilience*		1	(1.2%)
* M. avium* and *M. intracelullare* and *M. abscessus*		1	(1.2%)
* M. avium* and *M. absecessus* and *M. massiliense*		1	(1.2%)
Others	2	(2.4%)	
*M. chimaera and M. peregrinum*		1	(1.2%)
*M. gordonae and M. kyorinense*		1	(1.2%)

*Abbreviations*: *NTM* nontuberculous mycobacteria lung disease.

**Table 3:** **Bacterial colonization of respiratory tract among 84 patients with NTM lung diseases and 47 non**-**NTM BE patients**

	**NTM lung diseases patients (N = 84)**		**Non-NTM BE patients (N = 47)**		***P *** **value**
**Any isolates**	17	(20.2%)	12	(25.5%)	1.00
**Single isolate in one patient**	15	(19.7%)	12	(26.7%)	0.38
*Klebsiella pneumoniae*	4	(4.8%)	5	(10.6%)	0.28
*Streptococcus pneumoniae*	1	(1.2%)	1	(2.1%)	1.00
*Streptococcus species*, *viridans* group	1	(1.2%)	0		1.00
Methicillin-sensitive *Staphylococcus aureus*	1	(1.2%)	0		1.00
Methicillin-resistant *Staphylococcus aureus*	2	(2.4%)	0		0.54
*Pseudomonas fluorescens*	1	(1.2%)	0		1.00
*Pseudomonas putida*	1	(1.2%)	0		1.00
*Pseudomonas aeruginosa*	2	(2.4%)	5	(10.6%)	0.10
*Aeromonas hydrophila*	1	(1.2%)	1	(2.1%)	1.00
*Neisseria species*	1	(1.2%)	0		1.00
**Multiple microorganisms**	2	(2.4%)	0		0.54
Methicillin-sensitive *Staphylococcus aureus* and *Pseudomonas fluorescens*	1	(1.2%)	0		1.00
Methicillin-resistant *Staphylococcus aureus* and *Klebsiellapneumoniae*	1	(1.2%)	0		1.00

*Abbreviations*: *NTM* nontuberculous mycobacteria, *BE* bronchiectasis.

### Phenotypic characteristics of the participants

No difference in height was observed between NTM lung disease and non-NTM BE patients (160.0 *vs*. 159.0 cm, *P* = 0.23). However, patients with NTM lung disease were of lower body weight than those with non-NTM BE (54.0 *vs*. 55.5 kg, *P* = 0.04); consequently, the body mass index (BMI) of NTM lung disease patients was also lower than that of non-NTM BE patients (BMI = 20.8 *vs*. 22.2 kg/m^2^, *P* < 0.001). Scoliosis was more common among patients with NTM lung disease than those with non-NTM BE (23.8% *vs*. 8.5%, *P* = 0.04) (Table 4).

**Table 4:** **Phenotypic characteristics of 84 patients with NTM lung disease and 47 non**-**NTM BE patients**

	**NTM lung disease patients (N = 84)**	**Non-NTM BE patients (N = 47)**	***P *** **value**
Height (cm), median (IQR)	160.0 (156.0,168.0)	159.0 (153.0, 167.0)	0.23
Weight (kg), median (IQR)	54.0 (48.3, 60.0)	55.5 (51.0, 63.0)	0.04
BMI (kg/m^2)^, median (IQR)	20.8 (19.3, 22.1)	22.2 (20.5, 24.2)	<.001
Scoliosis, n (%)	20 (23.8%)	4 (8.5%)	0.04
Pectus excavatum*, n (%)	6 (7.1%)	0 (0.0%)	0.09

*Abbreviations*: *NTM* nontuberculous mycobacteria, *BE* bronchiectasis, *IQR* interquartile range. *Pectus excavatum was defined if the Haller index was > 3.5.

### Immunologic parameters of participants

Positive rheumatoid factor was detected more frequently in patients with non-NTM BE than with NTM lung disease (23.4% *vs*. 6.0%, *P* = 0.01); however, differences in mean rheumatoid factor levels were not statistically significant. No differences were seen regarding the presence of FANA, nor in FANA titers. Serum immunoglobulin levels were also similar between the two groups. Likewise, the proportion of IGRA-positive patients were similar in NTM lung disease patients and non-NTM BE patients (45.2 *vs*. 55.3%, *P* = 0.28) (Table 5).

**Table 5:** **Immunological parameters of 84 patients with NTM lung disease and 47 non**-**NTM BE patients**

		**NTM lung disease patients N = 84**	**Non-NTM BE patients N = 47**	***P *** **value**
Positive RF		5 (6.0%)	11 (23.4%)	0.01
RF level (0–9.9 IU/mL)^*^, median (IQR)		16 (15,92)	18 (15,86)	0.88
Positive FANA		11 (13.1%)	2 (4.3%)	0.14
FANA titer, median (IQR)		40 (40,50)	60 (40,80)	0.74
Positive IGRA		38 (45.2%)	26 (55.3%)	0.28
Patients with low level of immunoglobulins				
IgG	(700–1700 mg/dL)^*^	1 (1.2%)	0 (0%)	1.00
IgA	(90–400 mg/dL)^*^	3 (3.6%)	0 (0%)	0.55
IgM	(45–230 mg/dL)^*^	6 (7.1%)	4 (8.5%)	0.75
C3	(70–150 mg/dL)^*^	1 (1.2%)	1 (2.1%)	1.00
C4	(10–35 mg/dL)^*^	1 (1.2%)	1 (2.1%)	1.00

*Abbreviations*: *FANA* fluorescent antinuclear antibody test, *IGRA* IFN-γ release assay, *RF* rheumatoid factor, *NTM* nontuberculous mycobacteria, *BE* bronchiectasis, *IQR* interquartile range.

*Normal ranges.

### Radiological characteristics

The extent of BE was greater in the non-NTM BE group than in nodular bronchiectatic NTM lung disease patients (4 *vs*. 3 points, *P* = 0.001). Tubular BE was most common in both groups (Table 6).

**Table 6:** **Radiographic characteristics and extent of lesions of 84 patients with NTM lung disease and 47 non**-**NTM BE patients**

	**NTM lung disease patients (N = 84)**	**Non-NTM BE patients (N = 47)**	***P *** **value**
Extent of bronchiectasis^†^, median (IQR)	3 (2, 4)	4 (3, 9)	0.001
Type of bronchiectasis^‡^			
Tubular	46 (54.8%)	21 (44.7%)	0.28
Varicose	21 (25.0%)	11 (23.4%)	1.00
Cystic	17 (20.2%)	15 (31.9%)	0.14
Paranasal sinus			
Normal	62 (73.8%)	33 (70.2%)	0.69
Mucoepithelial thickening	21 (25.0%)	14 (29.8%)	0.55
Air-fluid level or total haziness	0 (0%)	0 (0%)	

*Abbreviations*: *NTM* nontuberculous mycobacteria, *BE* bronchiectasis, *IQR* interquartile range.

^†^Extent of bronchiectasis was scored in each 6 lobe according to involved percentage of bronchiectatic bronchi. Extent scores range from 0 to 18; 0 if less than 25%, 1 if 25 ~ 49%, 2 if 50 ~ 74%, 3 if 75% or more.

^‡^Classified based on main lesion.

### Pulmonary function

No differences in FVC were observed between NTM lung disease patients and non-NTM BE patients in terms of either absolute volume (2.8 *vs*. 2.7 L, *P* = 0.13) although percentage of predicted volume were smaller among non-NTM BE patients (94% *vs*. 87%, *P* = 0.02). Both absolute volume of FEV1 (2.1 *vs*. 1.8 L, *P* = 0.001) and percentage of predicted volume (101% *vs*. 86%, *P* = 0.001) were smaller among non-NTM BE patients. However, the proportion of patients who met criteria for chronic obstructive pulmonary disease was not different between two groups (13.6% vs. 23.9%, *P* = 0.15) (Table 7).

**Table 7:** **Pulmonary function of 81 patients with NTM lung disease and 46 non**-NTM BE patients*

**Number of patients**	**NTM lung disease patients (N = 81)**	**NonNTM BE patients (N = 46)**	***P *** **value**
FVC, L	2.8 (2.3,3.4)	2.7 (2.2,3.2)	0.13
% pred.	94 (81,104)	87 (76,97)	0.02
FEV_1_, L	2.1 (1.8,2.6)	1.8 (1.4,2.2.3)	0.001
% pred.	101 (85,112)	86 (67,99)	0.001
FEV_1_/FVC%	78 (72,82)	73 (65,81)	0.01
Patterns of ventilation			
Normal	52 (64.2%)	20 (43.5%)	0.03
Restrictive	16 (19.8%)	10 (21.7%)	0.82
Obstructive	11 (13.6%)	11 (23.9%)	0.15
Mixed	2 (2.5%)	5 (10.9%)	0.10

*The results of the pulmonary function test could not be performed because of bloody sputum or patients’ refusal in three NTM lung disease patients and one non-NTM-BE patient.

### Quality of life and emotional status

Median St. George’s respiratory questionnaire scores were similar between the two groups (19.5 *vs*.18.1, *P* = 0.34). Twenty patients (23.8%) with NTM lung disease and 10 patients (21.3%) with non-NTM BE reported anxiety (*P* = 0.83). Twenty-three 23 patients (27.4%) with NTM lung disease and 10 patients (21.3%) with non-NTM BE reported symptoms of depression (*P* = 0.53).

## Discussion

Through this prospective study, we collected phenotypic, immunologic, and other clinical data from patients with nodular BE type NTM lung disease and compared them with those of BE patients. These data confirmed several known characteristics of patients with NTM lung disease in the Korean population.

The phenotypic characteristics of patients with NTM lung disease in this study were similar to those described in previous reports [3,4,13,14]. Patients with NTM lung disease were leaner than non-NTM BE patients in our study. Although the underlying mechanism for the association between NTM lung disease and low BMI is not well understood, decreased leptin and increased adiponectin, and/or decreased estrogen in older women with low BMI may account for the increased susceptibility of these individuals to NTM infections [3]. In addition to low BMI, scoliosis and pectus excavatum are also frequently observed among NTM lung disease; a higher rate of scoliosis in patients with NTM lung disease was also seen in our study. These skeletal abnormalities may be indicative of an underlying genetic predisposition, though a precise mechanism linking the two has not been proposed [15].

*M. avium* complex and *M. abscessus* were the most common organisms isolated, consistent with a previous report on Korean patients with NTM lung disease [16]. Frequent isolation of *M. abscessus* is one of the characteristics of Korean patients that differ from patients from other countries [4]. Mixed infection by two or more NTM species was observed in 23.8% (20/84) of our patients; this observation confirmed our previous retrospective study showing high rates of mixed NTM infections [17]. Further examination will be necessary to determine the clinical significance of these mixed NTM infections.

We assessed several immunological markers, including FANA, rheumatoid factor, and serum immunoglobulins, and compared them with those of BE patients. Although most immunological markers were similar between the NTM lung disease and non-NTM BE groups, rheumatoid factor was found more commonly in patients with non-NTM BE. Given that the prevalence of rheumatoid arthritis and FANA were similar in both groups, the higher frequency of rheumatoid factor among patients with BE may be interpreted as a false positive. Various clinical settings have been shown to cause false positive results for rheumatoid factor [18].

NTM lung disease patients in our cohort were less likely to harbor *P. aeruginosa* than BE patients. Previous studies have also reported lower incidence of *P. aeruginosa* in BE or cystic fibrosis patients with NTM colonization. The underlying mechanism driving this phenomenon is not yet understood, but the observation that decontamination of *P. aeruginosa* yielded the cultivation of NTM colonization suggests a level of antagonism between *P. aeruginosa* and NTM [8,19]–[22].

A considerable number of NTM lung disease and non-NTM BE patients in our study reported feelings of anxiety (23.8% and 21.3%, respectively) as well as depression (27.4% and 21.3%, respectively). Given that the lifetime prevalence of depression and anxiety disorders in South Korea are 5.6% and 6.9%, respectively [23], rates reported here appear significantly higher than those in the general population. A similar study conducted in the United States showed that 20% of BE patients had elevated depression-related scores and 38% had elevated anxiety-related scores [24]. Consistently higher rates of depression and anxiety among NTM lung disease patients and non-NTM BE patients may stem from increased respiratory symptoms as well as other comorbidities.

## Conclusions

In conclusion, the characteristics of Korean NTM lung disease patients in this study were similar to those of patients in other countries. The fact that some phenotypic and clinical characteristics of NTM lung disease patients were different from those of non-NTM BE patients suggests differences in the immunopathogenesis of NTM lung disease and non-NTM BE.

## Competing interests

The authors declare that they have no competing interests.

## Authors’ contributions

Study concept and design: JJY. Acquisition of data: ARL, MWS, SAK, MK, KOC. Analysis and interpretation of data: ARL, JL, SMC, JJY. Drafting of the manuscript: ARL. Critical revision of the manuscript for important intellectual content: ARL, JL, SMC, MOS, JJY. Statistical analysis: ARL, JJY. Study supervision: JJY. All authors read and approved the final manuscript.

## Pre-publication history

The pre-publication history for this paper can be accessed here:

http://www.biomedcentral.com/1471-2334/13/558/prepub
